# Investigating critical brain area for EEG-based binocular color fusion and rivalry with EEGNet

**DOI:** 10.3389/fnins.2024.1361486

**Published:** 2024-02-27

**Authors:** Zhineng Lv, Xiang Liu, Mengshi Dai, Xuesong Jin, Xiaoqiao Huang, Zaiqing Chen

**Affiliations:** ^1^School of Information Science and Technology, Yunnan Normal University, Kunming, China; ^2^Engineering Research Center of Computer Vision and Intelligent Control Technology, Yunnan Provincial Department of Education, Kunming, China; ^3^Yunnan Key Laboratory of Optoelectronic Information Technology, Kunming, China; ^4^Information Network Center, The Second People’s Hospital of Yuxi, Yuxi, China

**Keywords:** binocular color fusion, binocular color rivalry, electroencephalography, EEGNet, BCI

## Abstract

**Introduction:**

Binocular color fusion and rivalry are two specific phenomena in binocular vision, which could be used as experimental tools to study how the brain processes conflicting information. There is a lack of objective evaluation indexes to distinguish the fusion or rivalry for dichoptic color.

**Methods:**

This paper introduced EEGNet to construct an EEG-based model for binocular color fusion and rivalry classification. We developed an EEG dataset from 10 subjects.

**Results:**

By dividing the EEG data from five different brain areas to train the corresponding models, experimental results showed that: (1) the brain area represented by the back area had a large difference on EEG signals, the accuracy of model reached the highest of 81.98%, and more channels decreased the model performance; (2) there was a large effect of inter-subject variability, and the EEG-based recognition is still a very challenge across subjects; and (3) the statistics of EEG data are relatively stationary at different time for the same individual, the EEG-based recognition is highly reproducible for an individual.

**Discussion:**

The critical channels for EEG-based binocular color fusion and rivalry could be meaningful for developing the brain computer interfaces (BCIs) based on color-related visual evoked potential (CVEP).

## 1 Introduction

With the improvement of various types of hardware and software performance, humans in the 21st century wanted to be able to restore a real world as much as possible in the monitor. Therefore, stereo display technology had become a hot topic of research for scholars (Liberatore and Wagner, 2021). Binocular color fusion and rivalry as two special phenomena in stereoscopic displays had also attracted a lot of attention and discussion. When the left and right eyes were viewing different colors at the same time, taking the red and the green as an example, if the color difference between red and green was small, the human brain can fuse the two colors as a single color. This phenomenon was called binocular color fusion. If the color difference between red and green increased to a certain threshold, the brain perceived periodic alternating changes of the red and the green. This phenomenon was called binocular color rivalry (Malkoc and Kingdom, 2012).

Binocular color fusion and rivalry, as two specific phenomena in binocular vision, reflected physiological changes in the brain’s visual perception of color. They could be used as experimental tools to study consciousness, attention, and how the brain processes conflicting information. A large number of studies had also been reported on the threshold values for binocular color fusion and rivalry. Jung et al. (2011) measured the binocular color fusion limit which was quantified by ellipses for eight chromaticity points sampled from the CIE 1976 chromaticity diagram. Malkoc and Kingdom (2012) measured the dichoptic color difference threshold (DCDT) in MacLeod-Boynton color space. The DCDT was the smallest detectable difference in color between two dichoptically superimposed stimuli. Chen et al. (2019) measured the binocular color fusion limit for five hues at different disparities in the 1976 CIE u’v’ chromaticity diagram. The binocular color fusion limit varied for each hue and different disparities. Xiong et al. (2021) conducted a psychophysical experiment to quantitatively measure the binocular chromatic fusion limit on four opposite color directions in the CIELAB color space. They suggested the fusion limit was independent of the distribution of cells and had nothing to do with the color inconsistency between eyes. The dominant eye might have some effects on binocular color fusion. But binocular color rivalry mainly involved the participation of brain cognition. However, these studies mainly used the subjective evaluation method to measure the binocular color fusion limit, and there was a lack of an objective indicator for judging binocular color fusion or binocular color rivalry.

The perception of binocular color fusion and rivalry in current traditional psychophysical experiments was mainly discriminated through subjects’ subjective reports, and lacked an objective judging index. And electroencephalography (EEG) was a technique for studying the relationship between brain activity and cognition, behavior, emotion, and physiological responses. It was also increasingly used by scholars to study the science of color. Cao et al. (2010) designed experiments to investigate the stimulus event-related potentials (ERPs) for blue/yellow colors in normal subjects. Experiments had shown that the brain was more sensitive to yellow in equal luminance mode. Yellow stimuli caused not only a shortening of the latency and an increase in the amplitude of the early ERP components N1 and P2, but also a shortening of the latency and an increase in the amplitude of the late ERP components N2 and P3. Wang and Zhang (2010) used red and blue car pictures to induce ERP. The experimental results showed that the average potential evoked by the red car picture was greater than that evoked by the blue car picture. Zakzewski et al. (2014) used eight different colors such as yellow, blue, red, cyan, white, black, magenta and green to design the experiment. Experiments were conducted to investigate whether EEG responses to stimuli of different colors were suitable for color classification. The results showed that only skewness data (averaged over 10 trials) could be used as a feasible feature for color classification by EEG features. Bekdash et al. (2015) designed experiments to study P1 components of normal subjects for four colors of different intensities. The four colors included red, green, blue, and yellow. The experimental results showed that the greater the color brightness, the greater the amplitude of P1 components. Liu et al. (2019) designed experiments to conduct the color-difference evaluation based on EEG signals. The experimental results showed that N1 component in the right-side and occipital area of brain had obvious regularity when observers were gazing at color-difference stimuli. However, the above studies did not target the discussion of the effect of individual variability on the EEG of binocular color fusion and rivalry as well as the experimental reproducibility for a single individual, which is the main issue explored in this paper.

EEG had become a key tool for research in many fields (Tang et al., 2022; Huang et al., 2023), but there were still some challenges in its analysis and processing. Firstly, the signal-to-noise ratio (SNR) of EEG was low (Jas et al., 2017). Second, individual variability among subjects also affected the performance of the model (Jeunet et al., 2016). Third, EEG statistics varied across time on the same individual (Gramfort et al., 2013; Cole and Voytek, 2019). To overcome the challenges described above, new approaches are required to improve the processing of EEG toward better generalization capabilities and more flexible applications. For example, the hierarchical nature of deep neural networks (DNNs) means that features can be learned from raw or minimally preprocessed EEG data, reducing the need for domain-specific processing and feature extraction pipelines. Features learned through DNNs might also be more effective or expressive than the ones engineered by humans (Roy et al., 2019). Deep learning (LeCun et al., 2015) was also gradually being applied to process EEG data in various fields (Bi et al., 2023) such as emotion recognition, epilepsy diagnosis, and depression diagnosis. Cecotti and Graser (2010) implemented the first classification of P300 event-related potentials using a Convolutional Neural Network (CNN). The method achieved a character recognition accuracy of 95.5%. Kulasingham et al. (2016) used a deep belief network and a stacked autoencoder to classify event-related potentials P300, achieving an average accuracy of 86.9% without feature extraction. Liu et al. (2018) introduced Batch Normalization into the training of network models to improve the performance for P300 classification. Liu et al. (2017) used SVM and Logistic classification model with EEG data of depressed patients as features while using CNN for recognition classification with 96.7% accuracy. Zheng and Lu (2015) used Deep Belief Networks to explore the key channels and bands for emotion recognition. Lawhern et al. (2018) proposed CNN-based EEGNet to accurately classify EEG signals for different brain-computer interface (BCI) paradigms. The above mainly described the research related to deep learning for emotion recognition, event-related potential classification, and depression diagnosis. However, for two special visual phenomena, binocular color fusion and rivalry, there were no studies using deep learning for recognition classification and exploration of key channels.

**Figure 1:**
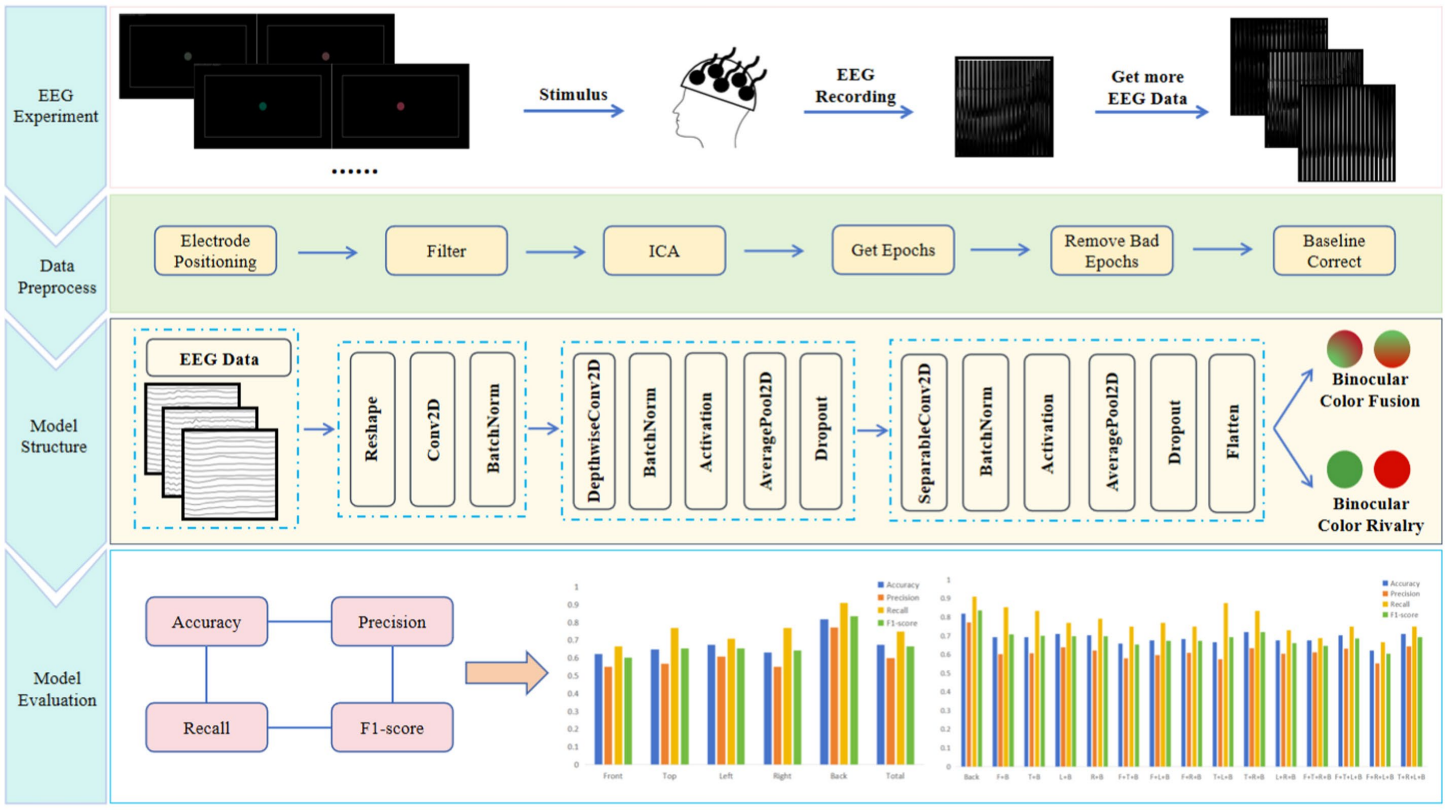
The general process of EEG-based classification for binocular color fusion and rivalry.

In this paper, we introduced EEGNet to construct an EEG-based model for binocular color fusion and rivalry classification. By using the model to explore the key channels for identifying binocular color fusion and rivalry, this research contributed to three distinct areas: 1, examining the model performance in five brain areas, investigating the critical brain area of brain associated with the binocular color fusion/rivalry task, 2, examining the model performance across subjects, exploring the effect of inter-subject variability on EEG-based judgmental recognition of binocular color fusion and rivalry, and 3, examining the model performance at a different time on the same individual, verifying the reproducibility across time with limited amounts of data. Figure 1 illustrates the general process of EEG-based classification for binocular color fusion and rivalry. The framework consists of four components: (1) the EEG experiment gives the specific design of our experiments and the specific process of data acquisition, (2) data preprocess describes our preprocess for the raw data, (3) model structure introduces the network framework of EEGNet, and (4) model evaluation presents the evaluation indicators and the model performance.

## 2 Materials and methods

### 2.1 Equipment and experimental environment

The EGI geodesic EEG system (GES 400) was used in this experiment. The GES 400 includes a standard electrode cap of 128 channels, an EEG signal amplifier, a computer with NetStation5 software and a computer with E-prime software. The computer with E-prime software was used to provide the stimulus images and provide event marks to the computer with NetStation5 software. Stimuli were displayed through a 23-inch size Samsung 3D monitor (S23A950D). The resolution of the monitor is 1920 (horizontal) * 1080 (vertical) pixels. The monitor offers a 2D/3D switching function with 3D glasses. The monitor connected to a graphics card (NVIDIA GeForce GTX 1080). To ensure that stimulus colors provided by the monitor are consistent with selected color samples, we used a PR-715 spectroradiometer to characterize the display appearing color through 3D glasses. The luminance of the digital input, as well as the luminance and chromaticity of the center point of the display, were obtained through the method of look-up-table (LUT). The computer with NetStation5 was used to collect and record the EEG signals transmitted by the electrode cap through the EEG signal amplifier.

To avoid the influence of other factors (especially external light), the whole experiments were conducted in a dark room. Subjects were required to minimize physical activity during the experiment to void the effect of electromyography (EMG) on the EEG data. According to the International Telecommunication Union standards (Series, 2009), subjects were required to sit approximately 860 mm from the screen to complete the experiment. Subjects were required to wear the electrode cap and 3D glasses connected to a monitor, which functioned by presenting different color stimulus images to the left and right eyes, thereby inducing binocular color fusion or binocular color rivalry.

### 2.2 Subjects

Ten university students participated in this experiment, all of whom had normal visual acuity, normal stereopsis and normal color vision, and their age range was 22–25 years. Subjects signed an informed consent form for the experiment, which met the criteria set out in the Declaration of Helsinki (WMA, 2022).

### 2.3 Stimuli

**Figure 2:**
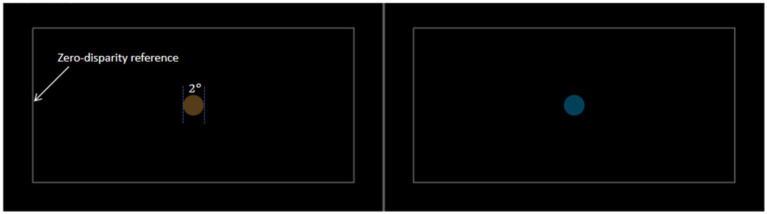
Example of color stimuli used in the experiment.

Stimulus images were generated by specially written software in C++, as shown in Figure 2. The size of the generated image was 3840*1080, and the subjects were presented with images of 1920*1080 size in the left and right eyes, respectively, by wearing 3D glasses. The color stimulus samples were selected in the CIELAB color space where luminance was fixed ( $L^{*}=30$ ). Subjects were presented with a color stimulus picture centered on a 2°circular block of color on a black background. Chen et al. (2019) had suggested that the gray rectangular boxes could be used as a zero-disparity reference to avoid or reduce the influence of disparity cues on the experimental result. The color values of the circular blocks were selected in the $a^{*}$ (red-green) and $b^{*}$ (yellow-blue) directions, and the specific color values were shown in Table 1. The left eye in Table 1 indicated the coordinates of the color sample points observed by the left eye. The right eye indicated the coordinates of the color sample points observed by the right eye. The selection of color sample points was based on our previous work (Xiong et al., 2021), which confirmed to induce binocular color fusion or rivalry for most normal people.

**Table 1:** Color stimulation values in the CIELAB color space.

Color stimulation type	Sample points pair			
	Left eye		Right eye	
	a*	b*	a*	b*
Fusion	0	−9	0	9
	−9	0	9	0
Rivalry	0	−24	0	24
	−24	0	24	0

### 2.4 Procedure

**Figure 3:**
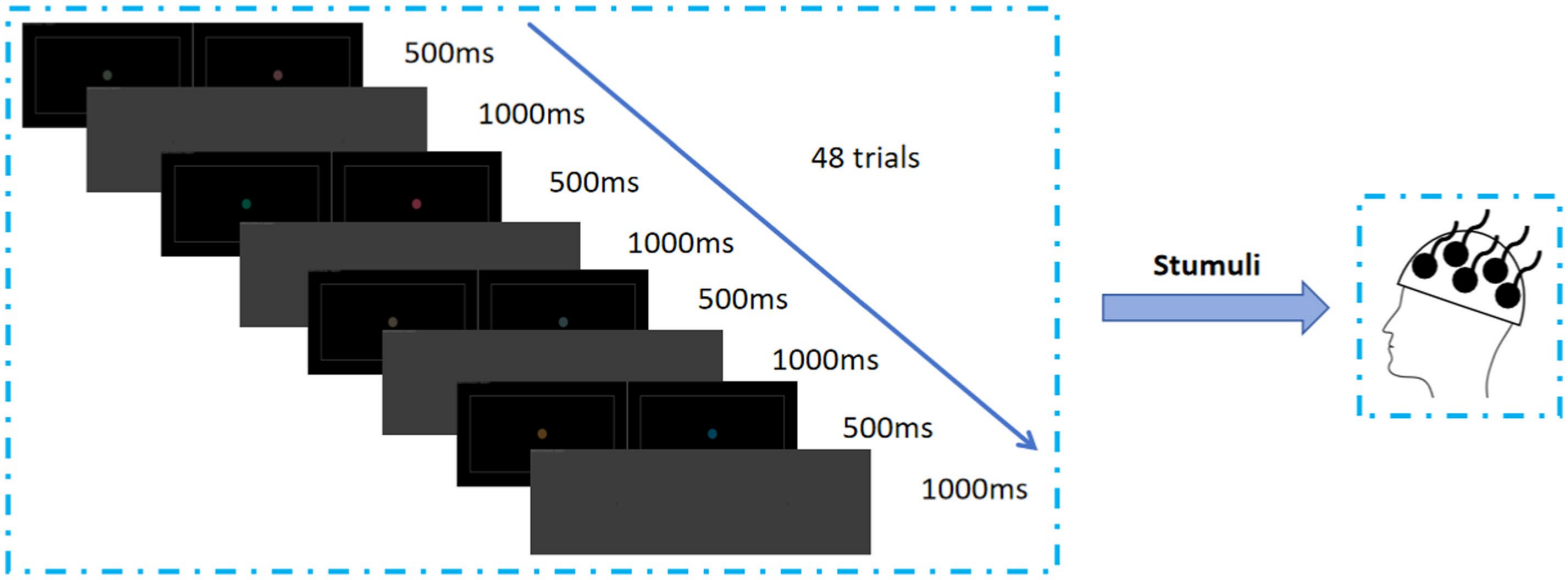
Presentation structure of test material.

There were four types of stimulus images: binocular color fusion on red/green direction (FoRG), binocular color rivalry on red/green direction (RoRG), binocular color fusion on yellow/blue direction (FoYB), binocular color rivalry on yellow/blue direction (RoYB). The experiment session did not last more than roughly 10 min at a time, including the explanations to the participant and 48 trials. The order of stimulus presentation for a trial was as follows: a FoRG image was presented for 500 ms, and a Mid-Gray field image lasted for 1,000 ms, and a RoRG image was presented for 500 ms, then the Mid-Gray field image was presented for 1,000 ms, next, a FoYB image was presented for 500 ms, and the Mid-Gray field image lasted for 1,000 ms, and a RoYB image was presented for 500 ms, and then Mid-Gray field image presented for 1,000 ms. The experimental stimulus presentation process was shown in Figure 3.

To explore the effect of inter-subject variability, the session was completed one time for eight subjects. Their experiments were conducted in the afternoon. To verify the reproducibility across time on the same individual, the session was completed 6 times for the other two subjects. Their experiments repeated 2 times in the morning, afternoon, and evening in 3 days. Hence, a total of 960 (= 8 subjects × 48 trials +2 subjects × 48 trials ×6 times) trials were recorded.

## 3 Results

### 3.1 Data preprocess

**Figure 4:**
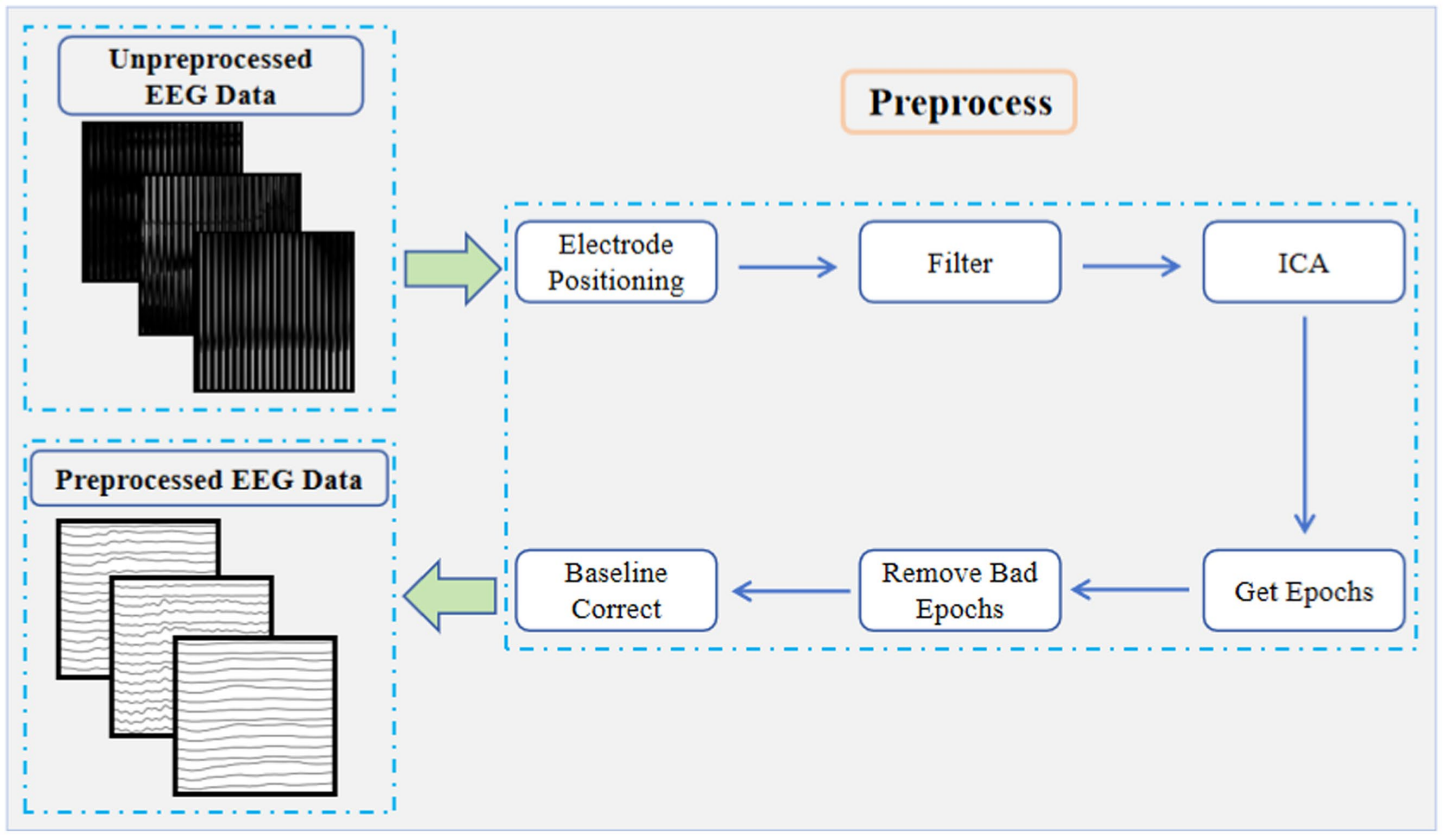
EEG data preprocessing flow.

We recorded the EEG data of the binocular color fusion/rivalry task for 10 subjects. Figure 4 gives the flow of EEG data preprocessing. The sampling rate of the raw data was 250 Hz. The experimental data were band-pass filtered from 0.5 Hz to 30 Hz. Then Independent Principal Component Analysis (ICA) was used to remove artifacts such as Electromyogram (EMG) and Electrooculogram (EOG) from the EEG data. After completing ICA, we extracted all the data from 200 ms before the color stimulus to 800 ms after the color stimulus. Each channel of the EEG data was divided into the same-length epochs without overlapping. EEG data from 200 ms before color stimuli were used as a baseline calibration and thresholds were designed to remove some extreme data (potential amplitudes greater than 100 μV). All preprocess was performed by writing code in Python MNE toolkit. Our raw data and source code were available at: https://figshare.com/s/e3350a7fb74e504012c7.

### 3.2 Model structure of EEGNet

**Figure 5:**
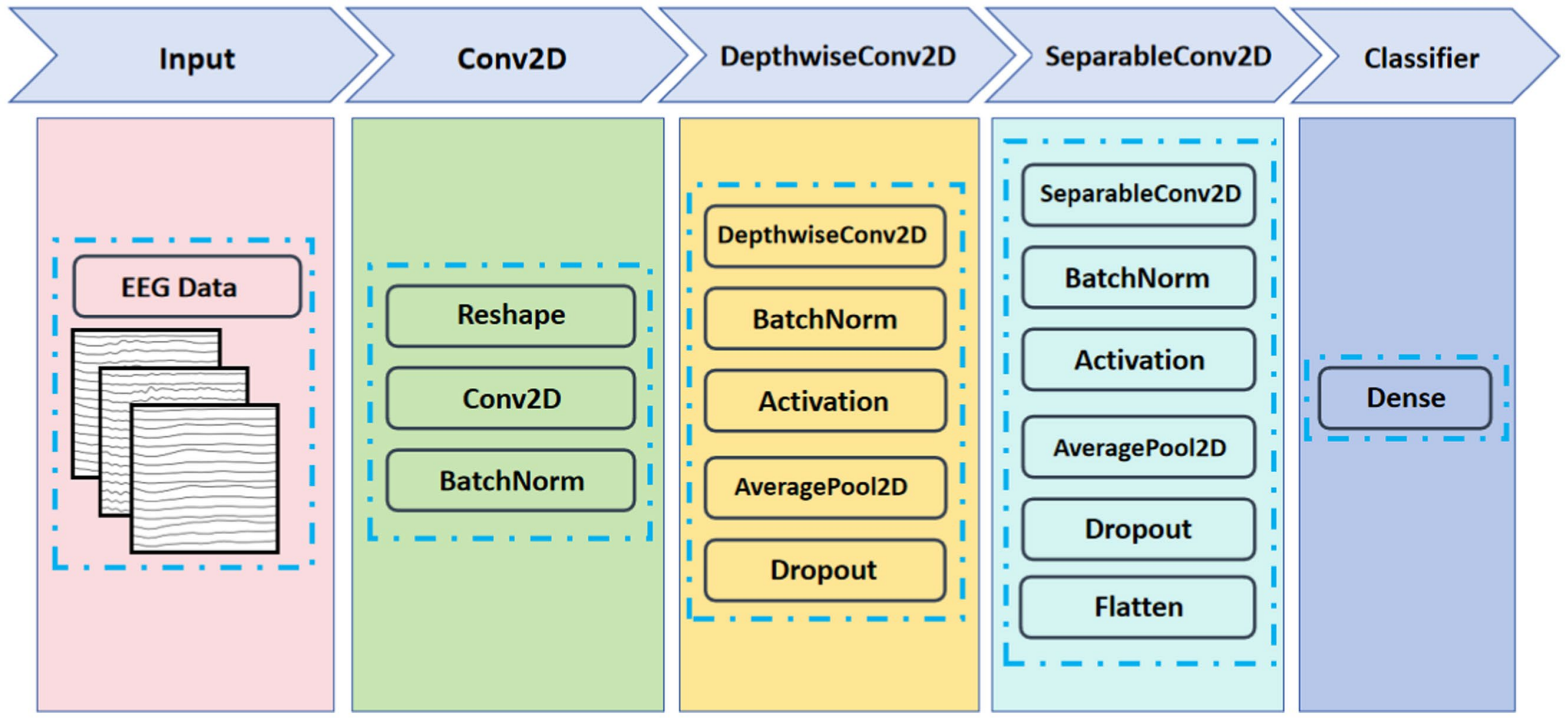
Architecture of EEGNet network.

EEGNet is a deep neural network-based EEG signal classification model (Lawhern et al., 2018). It can process raw EEG signals without complex feature extraction. The network structure of EEGNet mainly consists of a series of convolutional and pooling layers. Figure 5 shows the network structure of the deep neural network EEGNet. There are three types of convolution operations in the convolutional layer, including Conv2D, DepthwiseConv2D, and SeparableConv2D. Conv2D performs a convolution operation on EEG data in the time dimension to capture the temporal information and dynamic changes in the EEG signal. DepthwiseConv2D is used to process the spatial information of EEG signals. It computes the convolution independently for each channel of the input data and has a smaller number of parameters and computational complexity. Thus, DepthwiseConv2D can reduce the model parameters while maintaining a good feature extraction capability. DepthwiseSeparateConv2D consists of two parts, DepthwiseConv2D and SeparableConv2D. In the DepthwiseConv2D stage, it can be computed independently for each channel of the input data to capture the spatial information. And in the SeparableConv2D stage, the feature dimension is extended by applying a 1x1 convolution kernel to combine the channel information. This design allows a significant reduction in the number of parameters and improves the efficiency of the model. In EEGNet, SeparableConv2D is used to process the spatio-temporal information of EEG signals. Through the DepthwiseConv2D stage, the model is able to capture the spatial dependencies between different brain areas and extract distinguishing features. The SeparableConv2D stage increases the nonlinear expressiveness of the model and further enriches the feature representation.

In EEGnet, Batch Normalization (BN) operation is a normalization technique used to speed up the training process of neural networks and to improve the stability of the model. The BN operation focuses on normalizing the inputs for each layer in the neural network. The following expressions are the core formula of BN operation:


(1) $$\mu_{b}\leftarrow \frac{1}{m}\overset{m}{\underset{i=1}{\sum }}x_{i}$$

(2) $$\sigma_{b}^{2}\leftarrow \frac{1}{m}\overset{m}{\underset{i=1}{\sum }}{\left(x_{i}-\mu_{b}\right)}^{2}$$

(3) $${\hat{x}}_{i}\leftarrow \frac{x_{i}-\mu_{b}}{\sqrt{\sigma_{b}^{2}+\varepsilon }}$$

(4) $$y_{i}\leftarrow \gamma \;{\hat{x}}_{i}+\beta \text{≡}BN_{\gamma ,\beta }\left(x_{i}\right)$$

In Expression (1), $x_{1}$ ... $x_{m}$ are the first inputs, and $\mu_{b}$ is the mean $\mu_{b}$ of the data. In Expression (2), $\sigma_{b}^{2}$ is the variance of the data. Expression (3) is the most crucial, it is used to normalize the $x_{1}$ ... $x_{m}$ , and the main purpose of ϵ is to avoid the denominator being zero in the calculation of normalization. In Expression (4), the training related parameters γ , β and the output y are obtained by linear mapping of y to β to get the new data values. In forward propagation, the new distribution values can be derived from the learnable γ and β . At the time of backward propagation, the γ and β and the associated weights are derived by chaining the derivatives.

In EEGNet, Dropout is a common regularization technique used to reduce overfitting in neural networks. Specifically, two Dropout layers are used in EEGNet: one after DepthwiseConv2D and the other before the full connectivity layer. The role of the Dropout layer after the DepthwiseConv2D is to perform random discarding of the channel features at each time point. This helps the network to better learn the correlation between different channels and enhances the robustness of the network to noise and other disturbances. The Dropout layer before the fully connected layer serves to randomly discard the output of the full connectivity layer on each sample. This prevents the network from over-relying on some specific neurons and thus enhances the generalization ability of the network.

In this paper, the training can be briefly described as the following steps: (1) capture temporal features in the EEG data through Conv2D; (2) capture spatial features of the EEG through DepthwiseConv2D; and (3) use SeparableConv2D to continue to capture the channel feature information while reducing the number of parameters and computation, provide regularization, and finally pass the features directly to the Softmax classification unit.

### 3.3 Classifier training

We tried to use the deep neural network EEGNet to explore key areas of the brain about binocular color fusion and rivalry. We collected EEG data from 10 subjects, and all data was disrupted and reordered then put into EEGNet for training. Table 2 showed some of the training parameter information for the EEGNet. Dropout rate represented the random dropout ratio of Dropout. F1 was the number of filters in the DepthwiseConv2D; F2 was the number of filters in the SeparableConv2D. The number of samples in a batch at each iteration of training was 32; the optimizer was Adam; the loss function was CrossEntropyLoss; the activation function was ELU; the size of ϵ was 0.001; and the proportion of the test set was 10%.

**Table 2:** Training parameter information for the EEGNet.

Parameter	Dropout rate	F1	F2	Batchsize	Optimzer	Loss	Activation	ϵ	Test_size
Value	0.5	8	32	32	Adam	CrossEntropyLoss	ELU	0.001	0.1

### 3.4 Model performance in different areas

**Figure 6:**
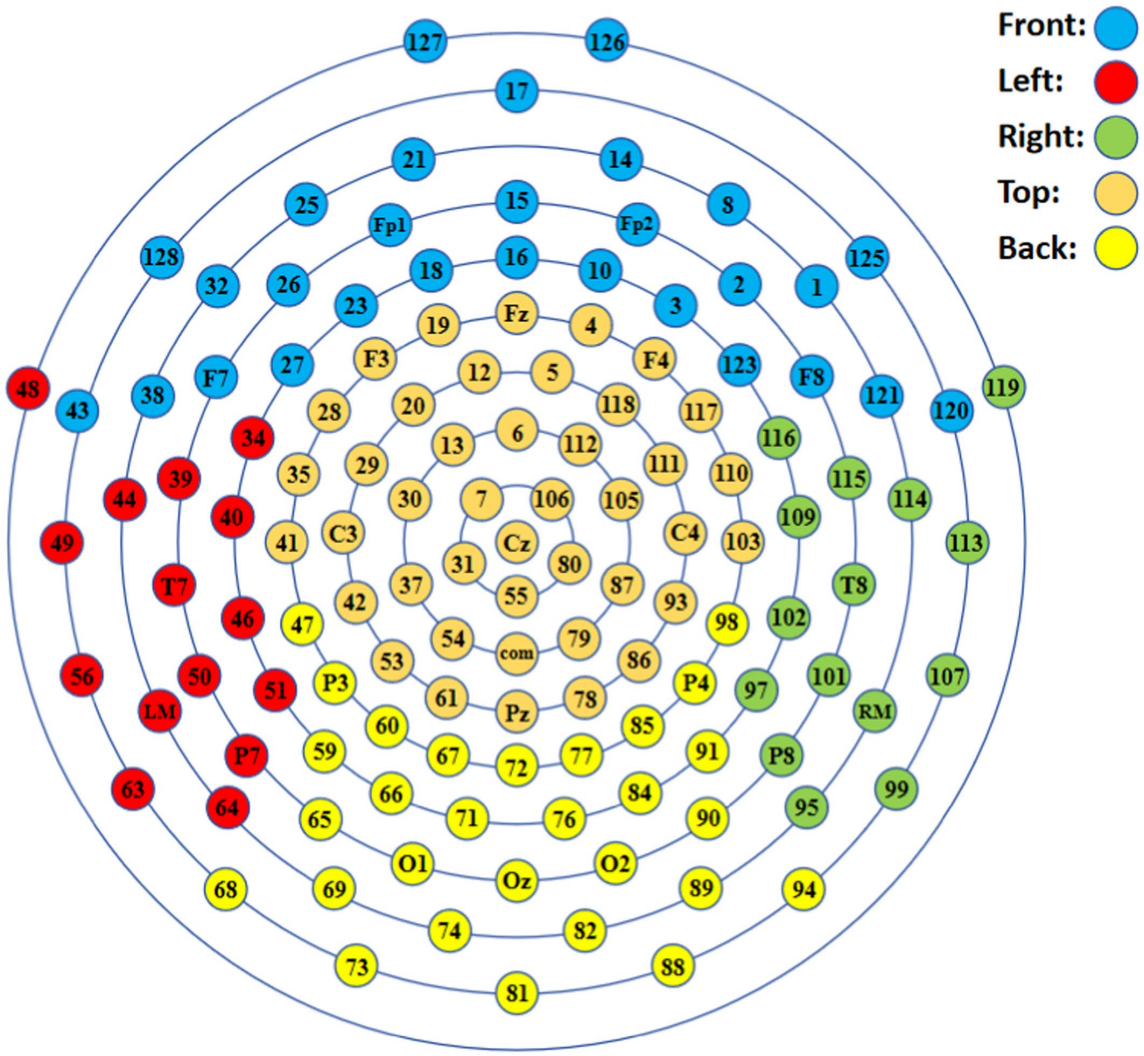
Division of electrode positions.

Identifying critical brain area for EEG-based binocular color fusion and rivalry is a matter for discussion. In this paper, we used the EGI geodesic EEG system (GES 400) to acquire multichannel EEG data up to 128 channels. Because of the large number of channels in the experimental data and the fact that the position of the electrode cap worn by each subject was to some extent affected by individual variability. We therefore envisaged dividing the electrode positions into five areas, according to the International 10–20 standard lead system (Sanei and Chambers, 2013), as shown in Figure 6. The front area (F) is concentrated in the prefrontal lobe, the left area (L) is distributed in the left temporal lobe, the right area (R) is distributed in the right temporal lobe, the top area (T) is in the parietal lobe, and the back area (B) covers the occipital region.

**Figure 7:**
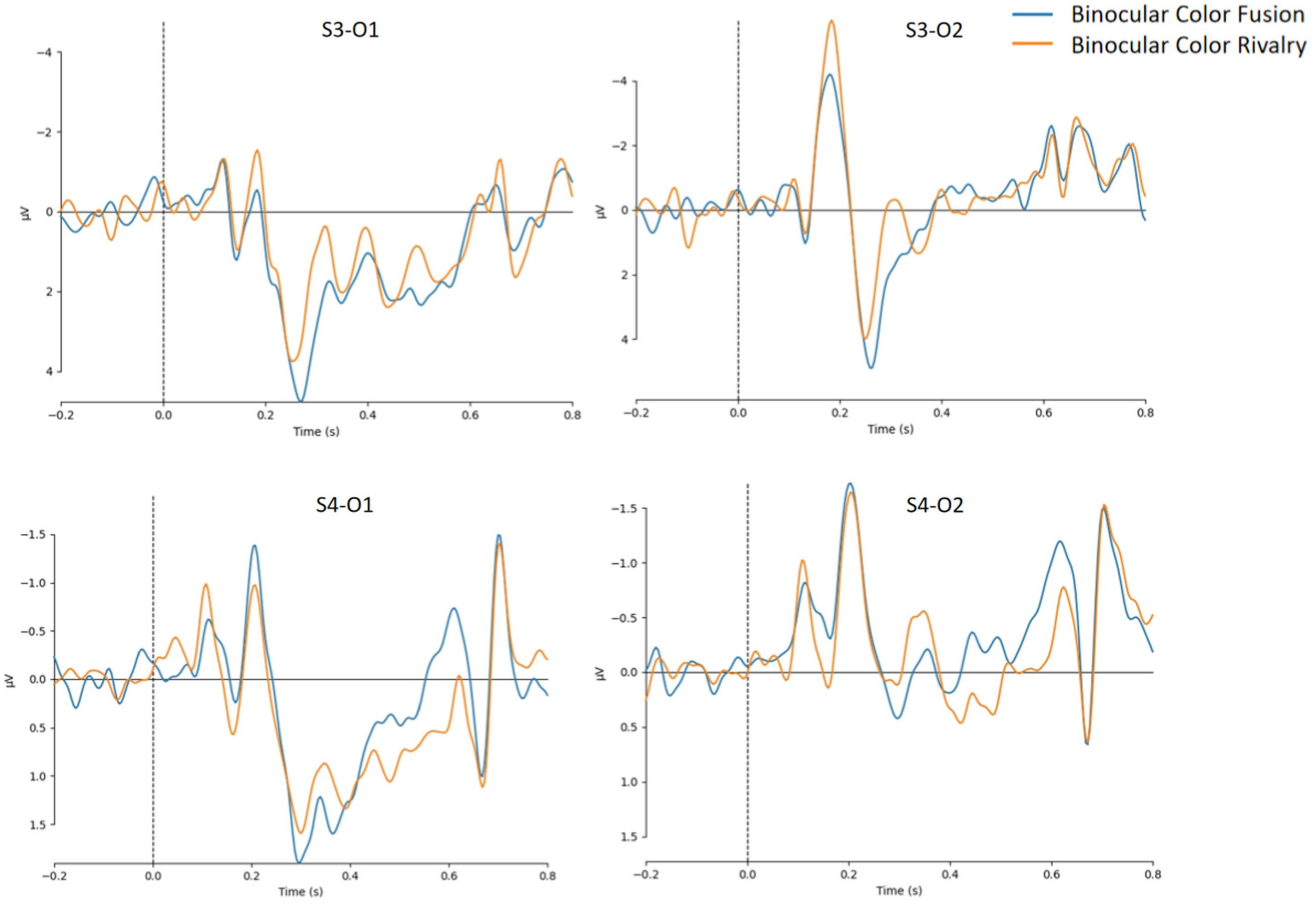
Mean potentials of O1, O2, and their surrounding 6 channels about binocular color fusion and rivalry for subject 3 (S3) and subject 4 (S4).

First, we trained models using EEGNet on corresponding data from 8 subjects (labeled S1-S8), and the indicators for model evaluation were shown in Table 3. For models trained on data from S1-S8, model performance was unsatisfactory for either the full 128 channels of data and each single area. The model did not effectively distinguish EEG data between binocular color fusion and rivalry. Based on the above experimental results, we then observed the average data of O1, O2, and their surrounding 6 channels for two subjects (S3 and S4), as shown in Figure 7. It can be seen from Figure 7 that there was a high degree of individual variability among the two subjects. This indicated that the poor performance of the EEGNet model might be due to the individual variability among subjects.

**Table 3:** EEGNet model evaluation indicators in single/full areas (S1-S8).

Area	Evaluation indicators			
	Accuracy	Precision	Recall	F1-score
Front	53.15	60.00	57.83	58.90
Top	55.94	65.15	51.81	57.72
Left	55.24	63.77	53.01	57.89
Right	44.76	53.13	40.96	46.26
Back	50.35	61.11	39.76	48.18
Full	55.94	64.29	54.22	58.82

**Figure 8:**
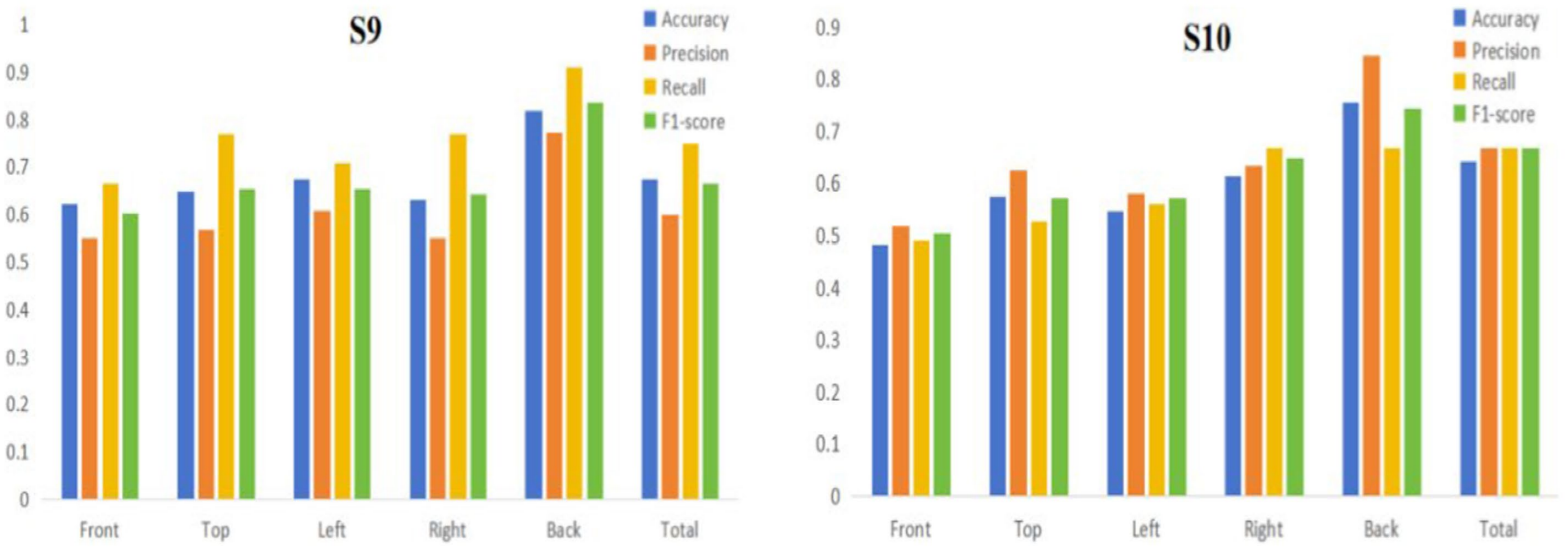
Histogram of EEGNet model evaluation indicators in single/full areas (S9, S10).

We then collected six sets of data for the other two subjects (labeled S9, S10) using the GES 400 to further verify whether there was an effect of individual variability between subjects on the performance of the EEGNet model and to validate the reproducibility of our experiments. We trained the model using EEGNet with the experimental data of the two subjects according to the previously divided areas, and the obtained model evaluation indicators are shown in Table 4 and Figure 8. By comparing the evaluation indicators of each model, we could find that for the same subject, the model trained with data from the back area could get relatively high evaluation indicators (Accuracy, Precision, Recall, and F1-score) compared to the model using data from full area or other single areas.

**Table 4:** EEGNet model evaluation indicators in single/full areas (S9, S10).

Area	Evaluation indicators (S9)				Evaluation Indicators (S10)			
	Accuracy	Precision	Recall	F1-score	Accuracy	Precision	Recall	F1-score
Front	62.16	55.17	66.67	60.38	48.11	51.85	49.12	50.45
Top	64.86	56.92	77.08	65.49	57.55	62.50	52.63	57.14
Left	67.57	60.71	70.83	65.38	54.72	58.18	56.14	57.14
Right	63.06	55.22	77.08	64.35	61.32	63.33	66.67	64.96
Back	**81.98**	**77.27**	**91.07**	**83.61**	**75.47**	**84.44**	**66.67**	**74.51**
Full	67.57	60.00	75.00	66.67	64.15	66.67	66.67	66.67

Bold numbers represent the maximum values of the model evaluation indicators in single/full areas.

**Figure 9:**
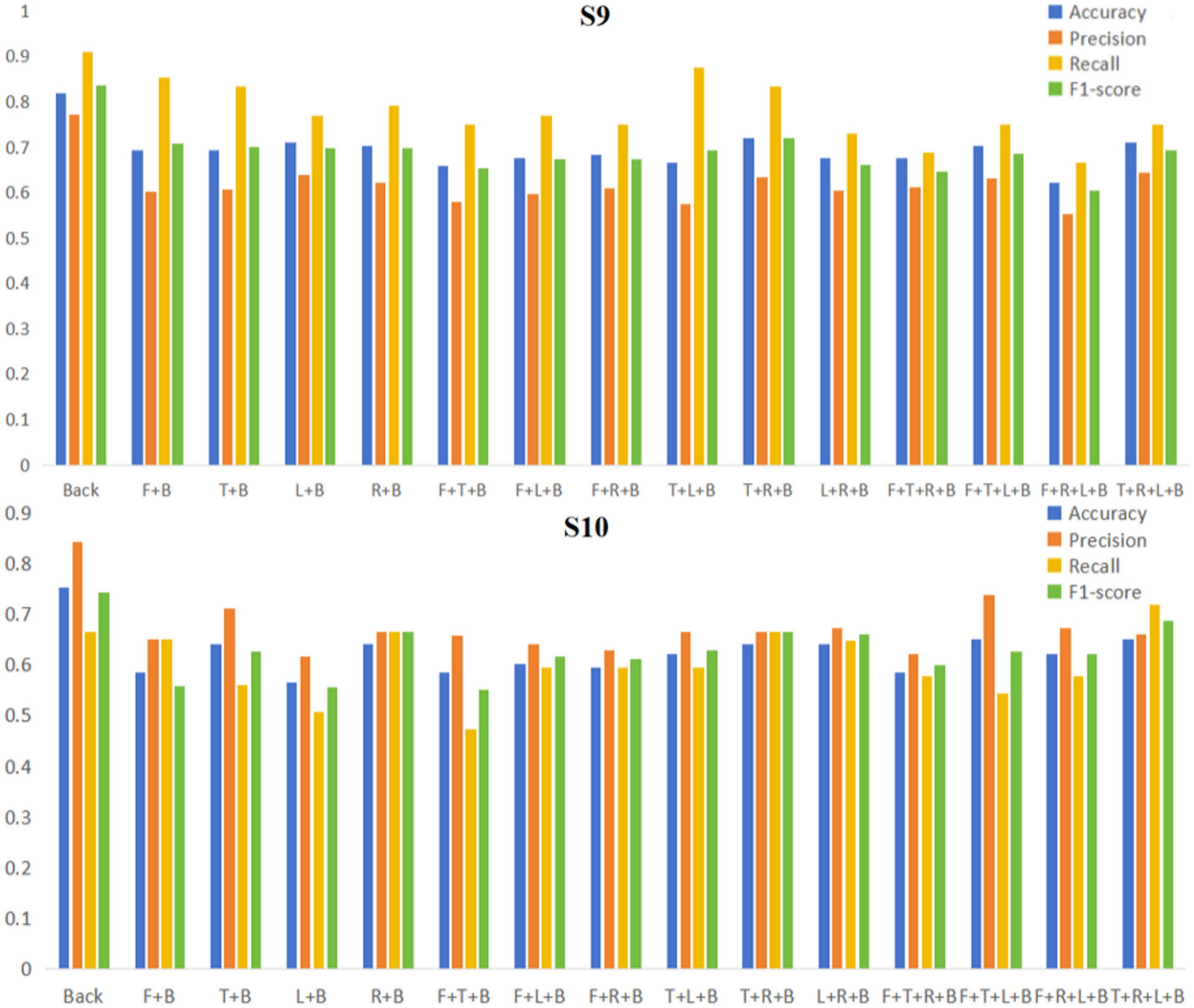
EEGNet model evaluation indicators in different regional combinations (S9, S10). F represents the Front area; T represents the Top area; L represents the Left area; R represents the Right area; and B represents the Back area.

To verify whether the back area is the critical brain area for EEG-based binocular color fusion and rivalry identification, we proceeded to train the model using EEGNet with the experimental data from the two subjects based on different combinations of areas (both containing the back area). The model performances were shown in Table 5 and Figure 9. The training results showed that for subject 9, all model evaluation indicators for the back area were higher than the combinations that included the back areas. For subject 10, the back area performed higher on three evaluation indicators than area combinations, except recall indicator of T + R + L + B combination. These results indicated that once data from other areas is added, the overall performance of the model actually decreases. Therefore, we can confirm that binocular color fusion and rivalry have a large difference in the data in the back area, and the back area is the key area for distinguishing binocular color fusion from rivalry.

**Table 5:** EEGNet model evaluation indicators in different regional combinations (S9, S10).

Area	Evaluation Indicators (S9)				Evaluation Indicators (S10)			
	Accuracy	Precision	Recall	F1-score	Accuracy	Precision	Recall	F1-score
F + B	69.37	60.29	85.42	70.69	58.49	65.12	65.12	56.00
T + B	69.37	60.61	83.33	70.18	64.15	71.11	56.14	62.75
L + B	71.17	63.79	77.08	69.81	56.60	61.70	50.88	55.77
R + B	70.27	62.30	79.17	69.72	64.15	66.67	66.67	66.67
F + T + B	65.77	58.06	75.00	65.45	58.49	65.85	47.37	55.10
F + L + B	67.57	59.68	77.08	67.27	60.38	64.15	59.65	61.82
F + R + B	68.47	61.02	75.00	67.29	59.43	62.96	59.65	61.26
T + L + B	66.67	57.53	87.50	69.42	62.26	66.67	59.65	62.96
T + R + B	72.07	63.49	83.33	72.07	64.15	66.67	66.67	66.67
L + R + B	67.57	60.34	72.92	66.04	64.15	67.27	64.91	66.07
F + T + R + B	67.57	61.11	68.75	64.71	58.49	62.26	57.89	60.00
F + T + L + B	70.27	63.16	75.00	68.57	65.09	73.81	54.39	62.63
F + R + L + B	62.16	55.17	66.67	60.38	62.26	67.35	57.89	62.26
T + R + L + B	71.17	64.29	75.00	69.23	65.09	66.13	**71.93**	68.91

F represents the front area; T represents the top area; L represents the left area; R represents the right area; and B represents the back area. Bold number represents the only model evaluation indicators in S9 and S10 that are larger than the Back area.

## 4 Discussion

For binocular color fusion and rivalry, above results and analysis demonstrated that the brain area represented by the back area (the occipital region) had a large difference on EEG signals, and more channels decreased the performance of the model. Reducing the number of electrodes reduced the computational complexity and also filtered out some irrelevant noise. Irrelevant channels require more computational cost and reduced the performance of the trained model. However, it should be noted that: although the performance of the model trained using the 29 channels of data from the back area is better than the performance of the model using the global channel data, this did not mean that the remaining 99 channels are useless for identifying binocular color fusion and rivalry.

In this study, our goal was to use EEGNet to validate the experiment reproducibility and to roughly determine the areas of the brain where binocular color fusion and rivalry is associated for different individuals. However, there are structural and functional differences in the brains of subjects. Different optimal combinations of channels may exist in different subjects. Some channels contribute significantly to the performance of some subjects, but not to others. Our results showed that there was a large effect of inter-subject variability on EEG-based judgmental recognition, and the statistics of EEG data is relatively stationary at different time on the same individual. Consequently, the EEG recognition of binocular color fusion and rivalry is still a very challenge across subjects, but the problem of across time could be solvable for an individual. We can train a model for each individual, to objectively judge the binocular color fusion and rivalry. And the individual model also can be used in BCI applications.

The rise of BCI in recent years has triggered scholars’ interest in EEG. As two special visual phenomena, binocular color fusion and rivalry are mostly judged based on the subjective evaluation of the subjects, and there is a lack of objective evaluation indexes. The current mainstream BCI paradigms are the steady-state visual evoked potential paradigm (SSVEP) (Vidaurre and Blankertz, 2010), the motor imagery paradigm (Xie et al., 2017), and so on. Chen et al. described how to use a BCI for high-speed spelling (Chen et al., 2015). The Berlin BCI group proposed a small-sample based motor imagery system (Vidaurre and Blankertz, 2010). Eye artifacts contaminating EEG signals were considered as a valuable source of information by Karas et al. (2023). The above studies involve different BCI paradigms, and for some of the already well-established BCI paradigms, the related key channels have been quite well studied. However, for some newly proposed BCI paradigms, these studies did not propose a simple and general method for exploring the key channels of a BCI paradigm. Offline analysis of EEG signals helps us to improve the classification accuracy, and once an EEG signal is classified, it can be sent to an external application for the rest of the operations. In addition to searching for key channels for binocular color fusion and rivalry, our experiments also performed classification operations on relevant EEG signals. This is very meaningful for the development of relevant BCI applications in the future. For example, the key channels of the relevant BCI paradigm are first found through our experimental approach, and then the optimal stimulus combinations are found to refine the previous BCI paradigm based on the classification results.

## 5 Conclusion

In this paper, we conducted EEG experiments to investigate critical brain area for binocular color fusion and rivalry classification with the EEGNet. By training the EEGNet model with data from 8 subjects (S1-S8) and comparing model performance for different channel combinations, we propose the hypothesis that individual variability between subjects has a great effect on the recognition of binocular color fusion and rivalry. By modeling two subjects (S9, S10) separately using EEGNet and comparing the model performance of different channel combinations, it is found that the model performance in the back area is higher than those in other areas. It helps us to verify that the individual variability between subjects has a large effect on the EEGNet model performance. It also verifies that our experiments are highly reproducible for the same individual. More importantly, the relatively high EEGNet model performance in the back area suggests that the back brain area is more distinct for binocular color fusion and rivalry. Reducing electrodes by selecting key channels not only reduces the cost of computation, but also significantly improves the performance and robustness of the model. This is of great research significance for the development of wearable devices for BCI based on the color-related visual evoked potential (VEP).

There are also some limitations to this study. The labels we consider in this study are limited to binocular color fusion and binocular color rivalry. In our future work, we will also apply the method proposed in this paper to more categories of datasets.

## Data availability statement

The datasets presented in this study ssssssscan be found in online repositories. The names of the repository/repositories and accession number(s) can be found at: https://figshare.com/s/e3350a7fb74e504012c7.

## Author contributions

ZL: Writing – original draft, Data curation, Methodology, Validation, Visualization. XL: Data curation, Formal analysis, Writing – review & editing. MD: Data curation, Formal analysis, Writing – review & editing. XJ: Data curation, Formal analysis, Writing – review & editing. XH: Data curation, Formal analysis, Writing – review & editing. ZC: Conceptualization, Project administration, Supervision, Writing – review & editing.
